# Childhood maltreatment as a risk factor for diabetes: findings from a population-based survey of Canadian adults

**DOI:** 10.1186/s12889-016-3491-1

**Published:** 2016-08-25

**Authors:** Margot E. Shields, Wendy E. Hovdestad, Catherine Pelletier, Jennifer L. Dykxhoorn, Siobhan C. O’Donnell, Lil Tonmyr

**Affiliations:** 1 Public Health Agency of Canada, 785 Carling Ave. 7th floor, Ottawa, ON K1A 0K9 Canada; 2 Public Health Agency of Canada, 9th Floor, room 9044, 1550 d’Estimauville 902-1550 d’Estimauville Ave, Quebec, G1J 0C5 Canada

**Keywords:** Child abuse, Child physical abuse, Child sexual abuse, Chronic disease, Exposure to intimate partner violence

## Abstract

**Background:**

It is well established that childhood maltreatment (CM) is a risk factor for various mental and substance use disorders. To date, however, little research has focused on the possible long-term physical consequences of CM. Diabetes is a chronic disease, for which an association with CM has been postulated.

**Methods:**

Based on data from a sample of 21,878 men and women from the 2012 Canadian Community Health Survey - Mental Health (CCHS - MH), this study examines associations between three types of CM (childhood physical abuse (CPA), childhood sexual abuse (CSA), and childhood exposure to intimate partner violence (CEIPV)) and diabetes in adulthood. Multiple logistic regression models were used to examine associations between CM and diabetes controlling for the effects of socio-demographic characteristics and risk factors for type 2 diabetes.

**Results:**

When controlling socio-demographic characteristics, diabetes was significantly associated with reports of severe and frequent CPA (OR = 1.8) and severe and frequent CSA (OR = 2.2). A dose–response relationship was observed when co-occurrence of CSA and CPA was considered with the strongest association with diabetes being observed when both severe and frequent CSA and CPA were reported (OR = 2.6). Controlling for type 2 diabetes risk factors attenuated associations particularly for CPA. CEIPV was not significantly associated with having diabetes in adulthood.

**Conclusion:**

CPA and CSA are risk factors for diabetes. For the most part, associations between CPA and diabetes are mediated via risk factors for type 2 diabetes. Failure to consider severity and frequency of abuse may limit our understanding of the importance of CM as a risk factor for diabetes.

## Background

It is well established that individuals who have experienced childhood maltreatment (CM) are at a higher risk for mental and substance use disorders in adolescence and adulthood [1, 2]. To date, however, little research has focused on the possible long-term physical consequences of CM, such as chronic conditions and diseases.

Diabetes is a chronic disease for which an association with CM has been postulated since many of the risk factors for type 2 diabetes (which represents 90–95 % of all diabetes cases in Canada [3]) are also associated with a history of CM. Therefore, any association between CM and diabetes may be mediated via risk factors for type 2 diabetes. For example, a recent meta-analysis found that CM was associated with a 40 % increase in the odds of developing obesity in adulthood [4], a common risk factor for type 2 diabetes [3, 5]. Similarly, CM has been shown to predict unhealthy behaviors in adolescence and adulthood such as smoking [6–9] and physical inactivity [7, 8], which are also risk factors for type 2 diabetes [3, 10, 11]. Finally, there is evidence that depression [12, 13] and hypertension [14, 15] are predictive of type 2 diabetes. Both have been shown to be outcomes of CM [1, 2, 16–18] and as such, may be in the causal pathway between CM and diabetes.

However, the few studies that have examined associations between CM and diabetes have yielded inconsistent results [7, 16, 19–23]. Various factors may account for these inconsistencies, including differences in the types of maltreatment being considered, measurement differences, whether or not severity and/or frequency are taken into account, the population studied, statistical approaches/methods, and power/sample size.

Based on data from a sample of 21,878 men and women from the 2012 Canadian Community Health Survey - Mental Health (CCHS - MH), this study examined associations between childhood physical abuse (CPA), childhood sexual abuse (CSA), and childhood exposure to intimate partner violence (CEIPV) and diabetes in adulthood. Furthermore, in light of findings from the literature [16, 19, 21], the co-occurrence of different forms of CM as well as severity and frequency of maltreatment were used to examine dose-response relationships between CM and diabetes. The role of type 2 diabetes risk factors (i.e., obesity, smoking, low physical activity level, high blood pressure, and lifetime history of depression) as mediators in the association between CM and diabetes was assessed. Since some studies have found gender differences in associations between CM and long-term health consequences [24–27], the analysis also tested for interactions between gender and CM in relation to diabetes. Finally, given that the prevalence of diabetes is higher among those aged 65 or older [3] and retrospective reports of CM tend to be lower among older versus middle-aged respondents [17], interactions between age (65 or older) and CM were also tested.

## Methods

### Data and sample

The 2012 CCHS - MH was conducted by Statistics Canada using a multistage stratified clustered sampling design [28]. The target population for the 2012 CCHS - MH was household residents aged 15 or older living in the 10 Canadian provinces. Excluded from the survey’s coverage were: persons living on reserves and other Aboriginal settlements; full-time members of the Canadian Forces; and the institutionalized population. Altogether, these exclusions represent about 3 % of the target population. The response rate was 68.9 %, yielding a sample of 25,113 individuals aged 15 or older [28].

CCHS - MH respondents were asked for permission to share the information they provided with Statistic Canada’s partners, which included the Public Health Agency of Canada. Most respondents (*n* = 23,709; 94 %) agreed to share. This article is based on data from only those respondents who agreed that their data could be shared.

The questions on CM were asked only of respondents aged 18 or older (*n* = 22,486). However, this study was based on a total sample of 21,878 as those respondents with missing values for CM (*n* = 412), diabetes (*n* = 5), or any of the socio-demographic or mediating variables (*n* = 191) were excluded.

### Measures

#### CM variables

**Fig. 1:**
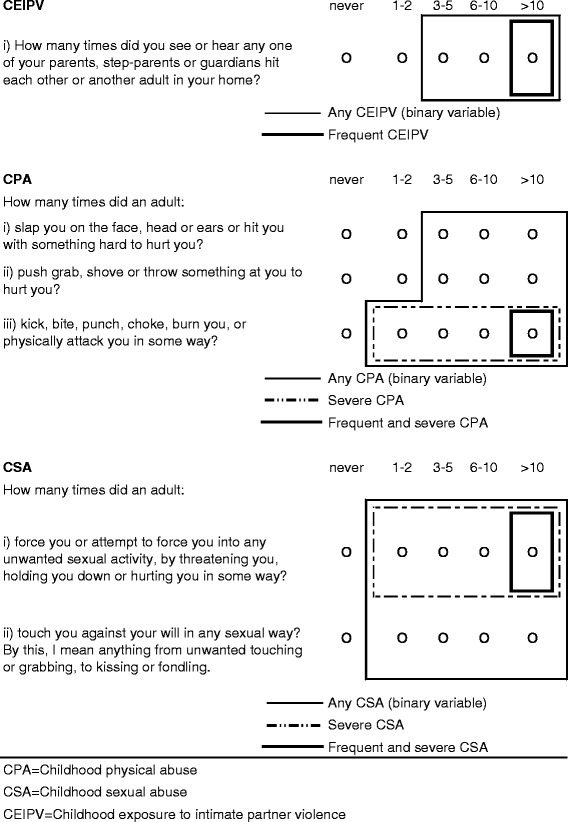
Child maltreatment items and definitions

CPA, CSA, and CEIPV were assessed by asking respondents about “*things that may have happened to you before you were 16 in your school, in your neighborhood, or in your family*” using the items shown in Fig. 1.

The items for CPA and CEIPV are from the Childhood Experiences of Violence Questionnaire (CEVQ), which has been shown to be reliable and valid in assessing maltreatment among youth in non-clinical settings [29]. The CSA items were very similar to ones previously used to assess adults’ experience of recent sexual violence in the 2009 General Social Survey [30].

For each type of abuse, binary variables (yes/no) were created following CEVQ guidelines [29]. Variables were also derived to indicate the severity and frequency of abuse as indicated in Fig. 1, similar to an approach previously used [21].

#### Diabetes

In the 2012 CCHS - MH, the presence of chronic conditions, including diabetes, was based on self-reported diagnosed illness. Respondents were asked about any “*long-term health conditions that have lasted or are expected to last six months or more and that have been diagnosed by a health professiona*l.” A checklist of conditions was included, one of which was diabetes. The question did not differentiate between type 1, type 2, and gestational diabetes.

#### Mediating variables

The role of five risk factors for type 2 diabetes were examined as potential mediators in the association between CM and diabetes. Note that risk factors for type 1 or gestational diabetes were not examined because these types constitute a minority (approximately 5 to 10 %) of cases of diabetes in Canada [3].

*Obesity* was assessed using *Body Mass Index* (BMI). Based on self-reported height and weight, BMI was calculated by dividing weight in kilograms (kg) by height in meters squared (m^2^). Correction factors were applied to adjust for known biases in self-reported BMI [31]. Using cut-points recommended by the World Health Organization [32], six categories were created based on corrected BMI (kg/m^2^) ranging from underweight (less than 18.5) to obese class III (40.0 or more).

*Smoking status* was divided into three categories: daily smoker, former daily smoker, never a daily smoker.

Based on the Canadian physical activity guidelines [33], respondents were classified as being *physically active* if they reported 150 min or more of moderate or vigorous physical activity in the past seven days.

*High blood pressure* was based on self-reported diagnosed illness and was included in the same checklist of chronic conditions as diabetes.

*Lifetime history of depression* was assessed using the World Health Organization version of the Composite International Diagnostic Interview and based on the criteria of the Diagnostic and Statistical Manual of Mental Disorders, 4^th^ edition [34].

Although findings have been inconsistent, some studies have suggested that stressful experiences in general may be associated with the onset of diabetes [35, 36]. Therefore, in supplementary analyses, perceived life stress (based on 5 categories) was included as a control variable in addition to the five mediating variables. In the CCHS – MH perceived life stress was assessed by asking respondents to think about the amount of stress in their lives and indicate if most days were not at all stressful, not very stressful, a bit stressful, quite a bit stressful or extremely stressful.

#### Socio-demographic characteristics

The socio-demographic characteristics included as covariates in the logistic regression models included *age* (used as a continuous variable), *sex, marital status* (married, widowed, divorced/separated, single/never married), *highest level of education attained by the respondent* (less than secondary graduation, secondary graduation, some postsecondary, postsecondary graduation), *household income* (quintiles based on household income adjusted by Statistics Canada’s low income cut-offs (LICO) specific to the number of individuals in the household, the size of the community, and the survey year), *immigrant status* (less than 20 years in Canada, 20 years or more in Canada, Canadian born), *ethnicity* (White, Black, Southeast/East Asian, off-reserve Aboriginal, other), *employment status* the week before the interview (employed, unemployed, not in the labour force), and *place of residence* (urban/rural).

### Analysis

Multivariate logistic regression analysis was used to examine associations between the CM variables and diabetes, controlling for socio-demographic characteristics (Model 1). Regressions were run based on the binary variables for CPA, CSA, and CEIPV as well as for the variables that accounted for severity and frequency of CM. Where significant associations were observed, the role of potentially mediating variables in associations between CM and diabetes was assessed by running five additional models; one model controlling for each of the five mediating variables (mediation models 2–6) in addition to the socio-demographic characteristics. A final model was run simultaneously controlling for all five mediating variables as well as the socio-demographic characteristics (mediation model 7).

To demonstrate mediation [37], we compared regression coefficients for the association between CM and diabetes before and after each mediator was added to the model. Since raw regression coefficients are not directly comparable across logistic regression models [38], the logistic regression coefficients were first standardized based on a formula proposed by Menard [39].

For each CM variable, the percentage change in the standardized regression coefficient is presented (i.e., the percentage change resulting from the inclusion of each mediator and all five mediators simultaneously). Attenuation in associations provides evidence of mediation [37]. Using standardized regression coefficients is beneficial when making comparisons of the effects of predictors across models since all predictors are converted to a common scale of measurement [39]. However, when demonstrating substantive findings for categorical variables, it is more relevant to present results based on unstandardized variables [39] and therefore odd ratios (ORs) are all based on unstandardized regression coefficients.

Analyses were conducted using SAS Enterprise Guide 5.1. All estimates are based on weighted data. Weights were created at Statistics Canada so that the data would be representative of the Canadian population living in the ten provinces in 2012 and were adjusted to compensate for non-response. Variance estimates and 95 % confidence intervals (CIs) were calculated using the bootstrap technique (with the SAS “proc survey” procedures) to account for the complex survey design of the 2012 CCHS – MH [28].

## Results

Diabetes was reported by 6.8 % of respondents (Table 1). The most commonly reported form of CM was CPA (26.1 %), followed by CSA (10.2 %), and then CEIPV (7.9 %). A prevalence of 2.6 % was observed for both severe and frequent CPA and severe and frequent CSA. When severity and frequency were taken into account, the correlation between CPA and CSA and between CSA and CEIPV was 0.29 and 0.27, respectively (data not shown). A strong correlation (0.44) was observed between CEIPV and CPA; among those who reported CEIPV, 76 % also reported CPA, among those who reported frequent CEIPV, 82 % also reported CPA (data not shown).

**Table 1:** Prevalence and sample sizes for diabetes and child maltreatment variables, household population aged 18 years or older, Canada, 2012

	Sample size	Weighted percent (95 % CI)
Total	21,878	
Diabetes		
Yes	1,837	6.8 (6.3, 7.3)
No	20,041	93.2 (92.7,93.7)
Childhood maltreatment variables		
CPA		
Yes	5,654	26.1 (25.1,27.2)
No	16,224	73.9 (72.8,74.9)
CPA severity and frequency		
Severe and frequent (>10 times) CPA	590	2.6 (2.2, 3.0)
Severe CPA (<=10 times)	1,666	7.1 (6.6, 7.7)
CPA (excluding severe CPA)	3,398	16.4 (15.5,17.3)
No CPA	16,224	73.9 (72.8,74.9)
CSA		
Yes	2,611	10.2 (9.6,10.9)
No	19,267	89.8 (89.1,90.4)
CSA: severity and frequency		
Severe and frequent (> = 3 times) CSA	682	2.6 (2.3, 3.0)
Severe CSA (<=2 times)	855	3.2 (2.9, 3.6)
Sexual touching (excluding severe CSA)	1,074	4.4 (4.0, 4.8)
No CSA	19,267	89.8 (89.1,90.4)
CEIPV		
Yes	1,762	7.9 (7.2, 8.5)
No	20,116	92.1 (91.5,92.8)
Frequency of CEIPV		
More than 10 times	938	4.3 (3.8, 4.8)
Three to 10 times	824	3.6 (3.2, 4.0)
Never, once or twice	20,116	92.1 (91.5,92.8)
Co-occurrence of CPA and CSA		
Both severe and frequent	164	0.6 (0.4, 0.7)
Either severe and frequent or both severe	1,153	4.8 (4.2, 5.4)
Either severe (neither frequent)	1,850	7.9 (7.4, 8.5)
Both CPA and CSA (neither severe)	275	1.2 (1.0, 1.4)
Either CPA or CSA (neither severe)	3,421	16.3 (15.4,17.2)
No CPA and no CSA abuse	15,015	69.2 (68.0,70.3)

Statistics Canada, Canadian Community Health Survey - Mental Health, 2012 (share file)

*CPA* Childhood physical abuse, *CSA* Childhood sexual abuse, *CEIPV* Childhood exposure to intimate partner violence, *CI* confidence interval

When controlling for age and sex, CPA (based on the binary variable) was not associated with diabetes (Table 2). However, when severity and frequency were taken into account, a significant association emerged for severe and frequent CPA (OR = 1.9).

**Table 2:** Adjusted odds ratios relating childhood physical abuse, childhood sexual abuse and childhood exposure to intimate partner violence to diabetes, household population aged 18 years or older, Canada, 2012

	Odds ratios controlling for age and sex	Odds ratios controlling for age, sex and socio-demographic factors
	Odds (95 % CI)	Odds (95 % CI)
CPA	1.1 (0.9, 1.3)	1.1 (0.9, 1.4)
CPA severity and frequency		
Severe and frequent (>10 times) CPA	1.9* (1.4, 2.7)	1.8* (1.3, 2.5)
Severe CPA (<=10 times)	1.3 (1.0, 1.7)	1.2 (0.9, 1.6)
CPA (excluding severe CPA)	0.9 (0.7, 1.1)	1.0 (0.8, 1.3)
No CPA (reference)		
CSA	1.5* (1.1, 2.0)	1.6* (1.2, 2.1)
CSA: severity and frequency		
Severe and frequent (> = 3 times) CSA	2.5* (1.4, 4.2)	2.2* (1.3, 3.7)
Severe CSA (<=2 times)	1.5 (1.0, 2.3)	1.6* (1.1, 2.4)
Sexual touching (excluding severe CSA)	1.0 (0.7, 1.5)	1.2 (0.8, 1.7)
No CSA (reference)		
CEIPV	1.3 (0.9, 1.9)	1.2 (0.9, 1.8)
Frequency of CEIPV		
More than 10 times	1.4 (0.9, 2.1)	1.3 (0.8, 2.0)
Three to 10 times	1.2 (0.8, 2.0)	1.2 (0.7, 2.0)
Never, once or twice (reference)		
Frequency of CEIPV (with no CSA)		
More than 10 times		
Three to 10 times	0.8 (0.3, 1.9)	0.7 (0.3, 1.8)
Never, once or twice (reference)	1.3 (0.5, 3.2)	1.2 (0.4, 3.0)
Co-occurrence of CPA and CSA		
Both severe and frequent		
Either severe and frequent or both severe	3.2* (1.7, 6.1)	2.6* (1.4, 4.9)
Either severe (neither frequent)	2.0* (1.4, 2.9)	1.9* (1.3, 2.8)
Both CPA and CSA (neither severe)	1.2 (0.9, 1.6)	1.3 (0.9, 1.7)
Either CPA or CSA (neither severe)	0.8 (0.4, 1.5)	0.9 (0.5, 1.8)
No CPA and no CSA abuse (reference)	0.9 (0.7, 1.1)	1.0 (0.8, 1.2)

Statistics Canada, Canadian Community Health Survey - Mental Health, 2012 (share file)

*CPA* Childhood physical abuse, *CSA* Childhood sexual abuse, *CEIPV* Childhood exposure to intimate partner violence, *CI* confidence interval

The odds ratios are based on unstandardized regression coefficients. The socio-demographic controls include age, sex, marital status, education, household income, immigrant status, ethnicity, employment status, and place of residence

*Significantly different from reference (*p* < 0.05)

The odds of reporting diabetes were 50 % higher when based on the binary variable for CSA. When severity and frequency were accounted for, those who reported severe and frequent CSA had 2.5 times the odds of reporting diabetes. For severe CSA that occurred less frequently (two or fewer time) the association with diabetes (OR = 1.5) only approached statistical significance (*p* = 0.054).

Based on the binary variable, CEIPV was not associated with diabetes. The odds ratio for frequent CEIPV and diabetes was somewhat elevated (1.4) but again non-significant. On account of the strong correlation between CEIPV and CPA, the association between CEIPV and diabetes was also examined excluding respondents who reported CPA; the odds ratio for frequent CEIPV was no longer elevated.

A dose-response relationship was observed when the co-occurrence of severe and frequent CPA and CSA was examined. Those who reported both severe and frequent CPA and CSA had 3.2 times the odds of reporting diabetes, those who reported either severe and frequent CPA or CSA or both severe (neither frequent) CPA and CSA, had twice the odds of reporting diabetes. The odds were slightly elevated (1.2), but not significant for those who reported either severe (neither frequent) CPA or CSA. Significant associations were not observed between less severe forms of CPA and CSA with diabetes.

When other socio-demographic factors were included in the models, all previously observed relationships persisted and the association between severe CSA occurring two or fewer times and reporting diabetes attained statistical significance.

### Effects of mediating variables

When risk factors for type 2 diabetes were included in the models to explore for potential mediating effects, the association between severe and frequent CPA and diabetes was attenuated (Table 3). The inclusion of obesity, smoking status, and high blood pressure resulted in reductions in the standardized regression coefficients of 13, 11, and 17 % respectively. The inclusion of all 5 mediators simultaneously resulted in a 41 % reduction, with the association between severe and frequent CPA and diabetes approaching statistical significance (*p* = 0.06).

**Table 3:** Adjusted odds ratios relating CPA to diabetes, household population aged 18 years or older, Canada, 2012

	Odds ratios controlling for socio-demographic factors:												
		and obesity		and smoking status		and physical activity level		and high blood pressure		and life-time depression		and diabetes risk factors	
	Odds (95 % CI)	Odds (95 % CI)	%∆	Odds (95 % CI)	%∆	Odds (95 % CI)	%∆	Odds (95 % CI)	%∆	Odds (95 % CI)	%∆	Odds (95 % CI)	%∆
CPA severity and frequency													
Severe and frequent (>10 times) CPA	1.8* (1.3, 2.5)	1.7* (1.2, 2.4)	−13	1.7* (1.2, 2.4)	−11	1.8* (1.3, 2.6)	−2	1.7* (1.2, 2.3)	−17	1.8* (1.3, 2.5)	−4	1.4 (1.0, 2.1)	−41
Severe CPA (<=10 times)	1.2 (0.9, 1.6)	1.2 (0.9, 1.6)		1.2 (0.9, 1.6)		1.3 (1.0, 1.7)		1.2 (0.9, 1.6)		1.2 (0.9, 1.6)		1.1 (0.8, 1.5)	
CPA (excluding severe CPA)	1.0 (0.8, 1.3)	0.9 (0.7, 1.2)		1.0 (0.7, 1.2)		1.0 (0.8, 1.3)		0.9 (0.7, 1.2)		1.0 (0.8, 1.3)		0.9 (0.7, 1.2)	
No CPA (reference)													
Diabetes risk factors													
Obesity - BMI category (range kg/m^2^)													
Underweight (less than 18.5)		0.6 (0.2, 2.5)										0.7 (0.2, 2.7)	
Normal weight (18.5 to 24.9) (reference)													
Overweight (25.0 to 29.9)		1.4* (1.0, 1.8)										1.3 (0.9, 1.7)	
Obese Class I (30.0 to 34.9)		2.5* (1.9, 3.2)										2.1* (1.5, 2.7)	
Obese Class II (35.0 to 39.9)		5.8* (4.2, 8.0)										4.2* (3.0, 5.8)	
Obese Class III (40.0 or more)		7.6* (5.3, 10.7)										5.1* (3.6, 7.3)	
Smoking status													
Daily smoker				1.2 (0.9, 1.6)								1.3 (1.0, 1.8)	
Former daily smoker				1.6* (1.4, 2.0)								1.5* (1.2, 1.8)	
Never a daily smoker (reference)													
Physically active						0.7* (0.6, 0.8)						0.8* (0.7, 0.9)	
High blood pressure								3.6* (2.9, 4.4)				2.8* (2.3, 3.5)	
Lifetime history of depression										1.2 (0.9, 1.5)		1.1 (0.9, 1.4)	

Statistics Canada, Canadian Community Health Survey - Mental Health, 2012 (share file)

*CPA* Childhood physical abuse, *CI* confidence interval

*%∆* percent change: Refers to the percent change in the standardized regression coefficient for child maltreatment variable resulting from the inclusion of mediating variables in the logistic regression model compared with the model only controlling for socio-demographic factors (only indicated if childhood maltreatment variable was significant in the socio-demographic model)

The odds ratios are based on unstandardized regression coefficients. The socio-demographic controls include age, sex, marital status, education, household income, immigrant status, ethnicity, employment status, and place of residence

*Significantly different from reference (*p* < 0.05)

For CSA, the effects of the mediating variables were not as pronounced (Table 4). The inclusion of obesity resulted in a reduction in the standardized regression coefficient for severe and frequent CSA and diabetes of 10 %. In the full model including all 5 mediators, the reduction was 17 %. For severe CSA occurring 2 times or less, the standardized regression coefficient was attenuated by 16 % with the inclusion of obesity and by 13 % with the inclusion of smoking status. In the full model, the reduction was 16 %, and the association with diabetes was no longer statistically significant.

**Table 4:** Adjusted odds ratios relating CSA to diabetes, household population aged 18 years or older, Canada, 2012

	Odds ratios controlling for socio-demographic factors:												
		and obesity		and smoking status		and physical activity level		and high blood pressure		and life-time depression		and diabetes risk factors	
	Odds (95 % CI)	Odds (95 % CI)	%∆	Odds (95 % CI)	%∆	Odds (95 % CI)	%∆	Odds (95 % CI)	%∆	Odds (95 % CI)	%∆	Odds (95 % CI)	%∆
CSA: severity and frequency													
Severe and frequent (> = 3 times) CSA	2.2* (1.3, 3.7)	2.2* (1.2, 3.9)	−10	2.1* (1.2, 3.5)	−7	2.2* (1.3, 3.7)	−1	2.2* (1.3, 3.9)	−4	2.2* (1.3, 3.7)	−4	2.0* (1.1, 3.7)	−17
Severe CSA (<=2 times)	1.6* (1.1, 2.4)	1.5 (0.9, 2.4)	−16	1.5 (1.0, 2.2)	−13	1.6* (1.1, 2.4)	2	1.6* (1.0, 2.6) 7		1.6* (1.0, 2.4)	−4	1.5 (0.9, 2.4)	−16
Sexual touching (excluding severe CSA)	1.2 (0.8, 1.7)	1.1 (0.8, 1.6)		1.1 (0.8, 1.6)		1.2 (0.8, 1.7)		1.2 (0.8, 1.7)		1.2 (0.8, 1.7)		1.1 (0.7, 1.6)	
No CSA (reference)													
Diabetes risk factors													
Obesity - BMI category (range kg/m^2^)													
Underweight (less than 18.5)		0.6 (0.2, 2.5)										0.7 (0.2, 2.7)	
Normal weight (18.5 to 24.9) (reference)													
Overweight (25.0 to 29.9)		1.4* (1.0, 1.8)										1.3 (0.9, 1.7)	
Obese Class I (30.0 to 34.9)		2.4* (1.9, 3.2)										2.0* (1.5, 2.7)	
Obese Class II (35.0 to 39.9)		5.8* (4.2, 8.0)										4.2* (3.0, 5.8)	
Obese Class III (40.0 or more)		7.5* (5.3, 10.6)										5.1* (3.5, 7.3)	
Smoking status													
Daily smoker				1.2 (0.9, 1.6)								1.3 (1.0, 1.7)	
Former daily smoker				1.6* (1.3, 2.0)								1.5* (1.2, 1.8)	
Never a daily smoker (reference)													
Physically active						0.7* (0.6, 0.8)						0.8* (0.7, 0.9)	
High blood pressure								3.6* (2.9, 4.4)				2.9* (2.3, 3.5)	
Lifetime history of depression										1.1 (0.9, 1.4)		1.0 (0.8, 1.3)	

Statistics Canada, Canadian Community Health Survey - Mental Health, 2012 (share file)

*CSA* Childhood sexual abuse, *CI* confidence interval

*%∆* percent change: Refers to the percent change in the standardized regression coefficient for child maltreatment variable resulting from the inclusion of mediating variables in the logistic regression model compared with the model only controlling for socio-demographic factors (only indicated if childhood maltreatment variable was significant in the socio-demographic model)

The odds ratios are based on unstandardized regression coefficients. The socio-demographic controls include age, sex, marital status, education, household income, immigrant status, ethnicity, employment status, and place of residence

*Significantly different from reference (*p* < 0.05)

When the mediating variables were included in the models examining the co-occurrence of severe and frequent CPA and CSA, there was some attenuation in the odds of reporting diabetes but the previously observed associations persisted (Table 5).

**Table 5:** Adjusted odds ratios relating CPA and CSA to diabetes, household population aged 18 years or older, Canada, 2012

	Odds ratios controlling for socio-demographic factors:												
		and obesity		and smoking status		and physical activity level		and high blood pressure		and life-time depression		and diabetes risk factors	
	Odds (95 % CI)	Odds (95 % CI)	%∆	Odds (95 % CI)	%∆	Odds (95 % CI)	%∆	Odds (95 % CI)	%∆	Odds (95 % CI)	%∆	Odds (95 % CI)	%∆
Co-occurrence of CPA and CSA													
Both severe and frequent	2.6* (1.4, 4.9)	2.8* (1.4, 5.5)	−2	2.4* (1.3, 4.4)	−3	2.6* (1.4, 5.0)	−2	2.3* (1.2, 4.4)	−23	2.6* (1.4, 4.7)	−2	2.3* (1.2, 4.4)	−22
Either severe and frequent or both severe	1.9* (1.3, 2.8)	1.8* (1.2, 2.7)	−17	1.8* (1.2, 2.6)	−10	1.9* (1.4, 2.8)	0	1.9* (1.3, 2.8)	−3	1.9* (1.3, 2.8)	−3	1.7* (1.1, 2.6)	−27
Either severe (neither frequent)	1.3 (0.9, 1.7)	1.2 (0.9, 1.7)		1.2 (0.9, 1.6)		1.3 (0.9, 1.7)		1.2 (0.9, 1.7)		1.2 (0.9, 1.7)		1.1 (0.8, 1.6)	
Both CPA and CSA (neither severe)	0.9 (0.5, 1.8)	1.0 (0.5, 1.8)		0.8 (0.4, 1.6)		0.9 (0.5, 1.8)		0.8 (0.4, 1.7)		0.9 (0.5, 1.8)		0.8 (0.4, 1.6)	
Either CSA or CSA (neither severe)	1.0 (0.8, 1.2)	0.9 (0.7, 1.2)		0.9 (0.7, 1.2)		1.0 (0.8, 1.3)		0.9 (0.7, 1.2)		1.0 (0.8, 1.2)		0.9 (0.7, 1.1)	
No CPA and no CSA (reference)													
Diabetes risk factors													
Obesity - BMI category (range kg/m^2^)													
Underweight (less than 18.5)		0.6 (0.2, 2.5)										0.7 (0.2, 2.7)	
Normal weight (18.5 to 24.9) (reference)													
Overweight (25.0 to 29.9)		1.4* (1.1, 1.8)										1.3 (0.9, 1.7)	
Obese Class I (30.0 to 34.9)		2.5* (1.9, 3.2)										2.1* (1.5, 2.7)	
Obese Class II (35.0 to 39.9)		5.8* (4.2, 8.0)										4.1* (3.0, 5.8)	
Obese Class III (40.0 or more)		7.5* (5.3, 10.7)										5.1* (3.6, 7.4)	
Smoking status													
Daily smoker				1.2 (0.9, 1.5)								1.3 (1.0, 1.7)	
Former daily smoker				1.6* (1.3, 2.0)								1.5* (1.2, 1.8)	
Never a daily smoker (reference)													
Physically active						0.7* (0.6, 0.8)						0.8* (0.7, 0.9)	
High blood pressure								3.6* (2.9, 4.4)				2.8* (2.3, 3.5)	
Lifetime history of depression										1.1 (0.9, 1.4)		1.0 (0.8, 1.4)	

Statistics Canada, Canadian Community Health Survey - Mental Health, 2012 (share file)

%∆ percent change: Refers to the percent change in the standardized regression coefficient for child maltreatment variable resulting from the inclusion of mediating variables in the logistic regression model compared with the model only controlling for socio-demographic factors (only indicated if childhood maltreatment variable was significant in the socio-demographic model)

*CPA* Childhood physical abuse, *CSA* Childhood sexual abuse, *CI* confidence interval

The odds ratios are based on unstandardized regression coefficients. The socio-demographic controls include age, sex, marital status, education, household income, immigrant status, ethnicity, employment status, and place of residence

*Significantly different from reference (*p* < 0.05)

In supplementary analyses, perceived life stress was included as a control variable in addition to the five mediating variables. In all cases the previously observed associations persisted (data not shown).

### Interactions with gender and age

Interactions between gender and CM were tested in all models. None of the interaction terms were significant. Significant interactions between age (65 or older) and CSA were observed but not for CPA or CEIPV. The interactions between CSA and age were negative, indicating a weaker association between CSA and diabetes for those aged 65 or older.

## Discussion

This study found a dose-response relationship between childhood CPA and CSA and diabetes in adulthood. The strongest association with diabetes was observed when both severe and frequent CPA and CSA were reported. CEIPV was not significantly associated with having diabetes in adulthood. Tests for gender and age differences in associations between CM and diabetes yielded non-significant results with the exception of CSA where a negative interaction was observed with age (65 years or older). This observed negative interaction between age and CSA could be due to under-reporting of CSA by older persons or premature mortality among victims of CSA [40].

Very few studies have examined associations between CM and diabetes using large population-based samples. Four studies based on relatively large samples used binary variables to measure CPA and CSA, not taking severity or frequency into consideration [7, 20, 22, 23]. Two of these studies, one based on close to 6000 participants from the US National Comorbidity Survey [20] and the other based on more than 9000 participants from a British birth cohort study [23] did not find significant associations for either CSA or CPA in relation to diabetes. A study based on 21,000 respondents aged 60 or older from Australia [7] found a significant association for CPA but not CSA. Similarly, a study based on more than 18,000 participants from ten countries [22] found a significant association for CPA but not CSA. These others studies did not have the multiple behavioural-specific questions used in the CCHS - MH and in some cases CM was based on a single subjective question (e.g., were you physically abused as a child?).

Similar to our approach, some studies have examined diabetes in relation to CM using abuse measures that account for severity and frequency. Using a large representative sample from the American population, Afifi [16] found a significant association between CPA (defined as responding “sometimes” or more frequent to having been hit so hard it left marks or bruises or caused injury) and diabetes but not with severe physical punishment (defined as responding “sometimes” or more frequent to how often a parent pushed, grabbed, slapped or hit you). Based on data for young adults aged 24–34 years from the National Longitudinal Study of Adolescent Health, Duncan et al. [19] found a significant association between recurrent CSA (> = 3 times) and diabetes for men but not for women and no association between CPA and diabetes for either sex. Results from a study using longitudinal data from the Nurses’ Health Study II [21] found a dose-response relationship between childhood CPA and CSA and incident type 2 diabetes similar to our findings. Mild CPA was not associated with diabetes risk, while moderate and severe CPA were associated with 26 and 54 % higher risk of incident diabetes. Unwanted sexual touching was associated with a 16 % higher risk of incident diabetes, one episode of forced sexual activity with a 34 % higher risk, and more frequent forced sexual activity with 69 % higher risk.

Similar to the study by Rich-Edwards [21], to some extent the associations between CPA and CSA and diabetes in our study were mediated by risk factors for type 2 diabetes. Adult obesity, smoking, and hypertension were important mediators in the association between CPA and diabetes. Controlling for all risk factors for type 2 diabetes simultaneously resulted in a 41 % reduction in the odds of reporting diabetes in relation to severe and frequent CPA and the association was no longer statistically significant. For CSA, there was less of a mediation effect when controlling for type 2 diabetes risk factors. Only adult obesity and smoking resulted in any appreciable reduction in the association between severe and frequent CSA and diabetes.

Another potential pathway that may explain the relationship between CM and type 2 diabetes is via the stress or trauma experienced by victims of CM. Results from clinical studies suggest that stressful experiences in early life result in frequent activation of the hypothalamic-pituitary-adrenal axis [41, 42]. This in turn can result in elevated cortisol levels and have lasting effects on the body’s stress-response system, including a heightened glucocorticoid, norepinephrine, and autonomic response [41, 43]. These changes can lead to insulin resistance, which in turn can cause increases in the blood glucose level, eventually resulting in type 2 diabetes [44]. Some studies have suggested that stressful experiences in general may be associated with the onset of diabetes [35, 36]. However, a meta-analytic review examining associations between adverse psychosocial factors (including stressful events) and diabetes found significant associations with the prognosis of diabetes but not incident diabetes [45]. In our study associations persisted when controlling for current perceived life stress, suggesting a unique association between CM and diabetes as opposed to an association with stress in general.

### Strengths and limitations

A major strength of this study is the large representative sample of Canadian adults. Also, the array of variables collected in the 2012 CCHS - MH made it possible to examine the mediating effects of several risk factors for type 2 diabetes and to control the potentially confounding effects of numerous socio-demographic factors when examining associations between CM and diabetes. In addition, the 2012 CCHS - MH included several CM questions making it possible to examine three types of CM in relation to diabetes as well as to consider the severity and frequency of maltreatment. Furthermore, the CCHS - MH CM items are behaviorally-specific and thus are likely to have higher validity and reliability than broad and subjectively defined items [46–49].

This study has some limitations that should be considered when interpreting results. All information collected was based on self-reports. A review of the literature on the validity of adult retrospective reports of adverse childhood experiences indicates that the rate of false negatives can be substantial, and that false positive reports are rare [47]. A study examining the psychometric properties of the CEVQ items concluded that it is a reliable and valid instrument with considerable agreement between self-reported CPA and CSA (including severe forms) and independent reports from clinicians [29]. Although the use of the behaviorally-specific CM items used in the 2012 CCHS - MH may have reduced the rate of false negatives in this study, the assessed types of CM may still have been underestimated due to recall bias. As well, it is possible that individuals who experienced CM who currently perceive themselves as being in good health are less likely to report the maltreatment. For CSA, items that separated *attempted* forced sexual activity from *actual* forced sexual activity would have allowed a more complete analysis of CSA severity.

In the 2012 CCHS - MH, respondents were asked to report on long-term health conditions lasting six months or more and that had been diagnosed by a health professional. Although misreporting could introduce bias, validity studies have found high agreement between self-reported diabetes and medical records [50, 51]. No information was collected about the specific type of diabetes (type 1, type 2, or gestational diabetes). However, previous studies have shown that that 90–95 % of diabetes cases in Canada are type 2 [3]. Finally, it is possible that some respondents may have had diabetes that had not yet been diagnosed by a health care professional. A study based on plasma glucose readings using data from the 2007 to 2009 Canadian Health Measures Survey found that 0.9 % of the Canadian population aged 6 or older had undiagnosed diabetes, representing 20 % of all cases of diabetes [3].

It is unknown how these limitations of the diabetes and CM measures might influence associations. Furthermore, it is possible that use of more objective measures of the risk factors for type 2 diabetes (e.g., measured BMI) might result in further attenuations in associations between CPA/CSA and diabetes. As well, family history of diabetes and abnormal lipid profile were not measured and could account for some residual variance.

The cross-sectional nature of the data precludes establishing the temporal order of events and conclusions regarding the causal nature of associations. However, a study comparing the associations between CM and adverse health outcomes in adulthood concluded that retrospective and prospective studies yield similar results [52]. When testing for mediation, it is assumed that the mediation variable is in the pathway between CM and the diagnoses of diabetes. This may not always be the case. For example, the association between depression and diabetes is complex; some studies have found depression is associated with incident type 2 diabetes [12, 13], while others have found that type 2 diabetes precedes depression [12, 53]. If the latter is true, it would be inappropriate to consider depression as a mediating variable.

Finally, the degree to which findings in this study may be attributable to unmeasured factors such as childhood socioeconomic status and other childhood family adversities such as neglect, emotional abuse, and parental mental and substance abuse disorders is unknown. However, analysis of the data from the Nurses’ Health Study II considered several early childhood covariates as potential confounders (including birth weight, parental history of diabetes, and parental education and occupation) and the observed associations between CM and diabetes persisted [21].

### Implications

Diabetes is the sixth leading cause of death in Canada [54] and reduces the health-related quality of life for those living with the disease [55]. Individuals with diabetes are at risk for a number of long-term and life-threatening complications including heart disease, stroke, blindness, kidney disease, and lower-limb amputation [56]. Based on the results from this study, associations between CM and diabetes were the strongest for repeated and severe incidents of childhood CPA and CSA. Failure to consider severity and frequency of CPA and CSA may limit our understanding of the importance of CM as a risk factor for diabetes. Early intervention is critical to reduce the risk that people who have experienced CM will develop this debilitating disease.

## Abbreviations

BMI: Body mass index
CCHS–MH: Canadian Community Health Survey-Mental Health
CEIPV: Childhood Exposure To Intimate Partner Violence
CI: Confidence Interval
CM: Childhood Maltreatment
CPA: Childhood Physical Abuse
CSA: Childhood Sexual Abuse
OR: Odds Ratio
