# Erratum to: Childhood maltreatment as a risk factor for diabetes: findings from a population-based survey of Canadian adults

**DOI:** 10.1186/s12889-016-3754-x

**Published:** 2016-10-14

**Authors:** Margot E. Shields, Wendy E. Hovdestad, Catherine Pelletier, Jennifer L. Dykxhoorn, Siobhan C. O’Donnell, Lil Tonmyr

**Affiliations:** 1 Public Health Agency of Canada, 785 Carling Ave. 7th floor, Ottawa, ON K1A 0K9 Canada; 2 Public Health Agency of Canada, 9th Floor, room 9044, 1550 d’Estimauville 902-1550 d’Estimauville Ave, Quebec, G1J 0C5 Canada

## Erratum

Upon publication of this article [1], it was brought to our attention that Fig. 1 contained an error under heading CSA, question “How many times did an adult: i) force you or attempt to force you into any unwanted sexual activity, by threatening you, holding you down or hurting you in some way?”.

**Fig. 1:**
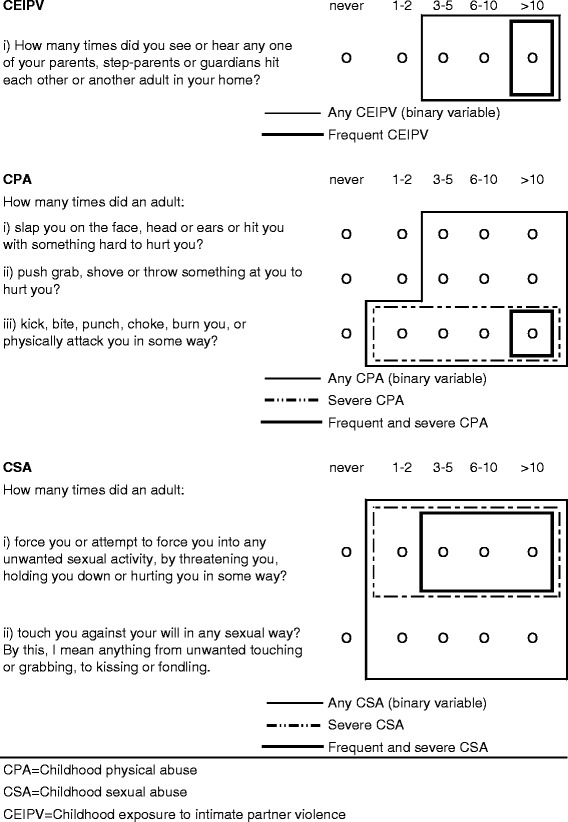
Child maltreatment items and definitions

The thick frame in CSA should encompass not only the >10 bubble, but also the 3–5 and 6–10 bubbles, as shown in modified Fig. 1.

Figure 1 was updated in the original article.
